# Knockdown of IER3 Promotes Osteogenic Differentiation of Human Mesenchymal Stem Cells

**DOI:** 10.3390/biomedicines13040947

**Published:** 2025-04-12

**Authors:** Yuqing Han, Hongyang Ma, Zhihui Tang, Chanyuan Jin

**Affiliations:** Second Clinical Division, Peking University School and Hospital of Stomatology & National Center of Stomatology & National Clinical Research Center for Oral Diseases, Beijing 100081, China; yuqinghanyq@163.com (Y.H.); hongyang.ma@pku.edu.cn (H.M.)

**Keywords:** human mesenchymal stem cells, IER3, osteogenic, MAPK/ERK signaling, differentiation

## Abstract

**Background**: The differentiation process of human mesenchymal stem cells (hMSCs) is regulated by a variety of chemical, physical, and biological factors. These factors activate distinct signaling pathways and transcriptional networks, thereby regulating the lineage-specific differentiation of hMSCs. **Objective**: This study aims to investigate the role of Immediate Early Response 3 (IER3) in the osteogenic differentiation of human mesenchymal stem cells (hMSCs) and explore the underlying regulatory mechanisms by which IER3 influences osteogenesis. **Methods**: The expression levels of IER3 and osteogenesis-related genes were quantified when hMSCs were subjected to in vitro osteogenic induction. Then, stable IER3–knockdown hMSCs were generated using IER3–targeted shRNA lentiviral vectors, and the impact of IER3 on osteogenic differentiation was evaluated through both in vitro cell induction and hMSCs subcutaneous implantation model of nude mice. Moreover, RNA–seq and functional inhibition assays were performed to elucidate the signaling pathway through which IER3 regulates the osteogenic differentiation of hMSCs. **Results**: IER3 expression was significantly downregulated during osteogenic differentiation. Knockdown of IER3 markedly upregulated the expression of ALP and RUNX2, enhancing the osteogenic differentiation capacity of hMSCs, both in vitro and in vivo. Mechanistic studies revealed that IER3 knockdown significantly increased phosphorylated ERK1/2 levels, activating the MAPK/ERK signaling pathway. Furthermore, inhibition of the MAPK/ERK signaling pathway reversed the enhanced osteogenic differentiation observed following IER3 knockdown. **Conclusions**: Knockdown of IER3 promotes osteogenic differentiation of hMSCs through regulation of the MAPK/ERK signaling pathway, indicating IER3 represents a potential therapeutic target for the treatment of osteoporosis and bone defect-related diseases.

## 1. Introduction

With the global trend of population aging, the incidence of bone-related diseases such as osteoporosis and bone defects has been increasing year by year, posing a serious threat to human health and imposing a significant economic burden on healthcare systems worldwide [1]. Traditional treatment methods, including pharmacological interventions and surgical repairs, have limitations such as drug side effects, surgical trauma, and high costs [2,3]. Therefore, exploring new therapeutic strategies for bone-related diseases has become a focal point in medical research. In recent years, the continuous development of bone tissue engineering has provided new insights into the treatment of bone-related diseases, with stem cell therapy gaining attention due to its unique tissue repair capabilities and demonstrating broad clinical application prospects [4,5].

Mesenchymal stem cells (MSCs) are a subset of stem cells with the ability to self-renew and differentiate [6]. In 1966, Friedenstein et al. first identified MSCs in bone marrow, and, since then, extensive research using both in vitro and in vivo models has expanded our understanding of MSCs [7]. Under appropriate stimuli, MSCs can differentiate into various tissue-specific cell types, facilitating tissue repair and playing a significant role in disease treatment [8,9]. The differentiation process of MSCs is regulated by various chemical, physical, and biological factors, which activate distinct signaling pathways and transcription factors to direct lineage-specific differentiation [10,11]. During the osteogenic differentiation of MSCs, the transcription factor RUNX2 and the osteogenic marker alkaline phosphatase (ALP) are generated early to induce matrix production. In the later mineralization phase, osteocalcin (OCN) is secreted by mature osteoblasts [12,13,14]. The bone morphogenetic protein (BMP) pathway, Wnt/β-catenin pathway, and Notch pathway are the primary signaling pathways involved in MSCs osteogenic differentiation [15,16,17]. Additionally, classic signaling pathways, such as MAPK/ERK and PI3K/AKT, contribute to the regulation of MSCs osteogenesis and adipogenesis [18]. Given their key role as a primary source of osteoblasts, understanding the mechanisms of MSC osteogenic differentiation is crucial in research on osteoporosis and bone defect-related diseases.

Immediate Early Response 3 (IER3), also known as Immediate Early X gene 1 (IEX-1), is induced by various stimuli, including growth factors, cytokines, ionizing radiation, viral infections, and other types of cellular stress [19]. IER3 is primarily localized in the cytoplasm and nucleus. Its localization may vary depending on cell type [20,21]. Human IER3 comprises 156 amino acids, and all orthologues structurally share a putative transmembrane domain, a potential glycosylation site, a Ser/Thr phosphorylation site near the N-terminus, and a nuclear localization site (NLS) [19]. IER3 plays an important role in cellular stress response, apoptosis, and proliferation. IER3 plays a dual role in cell proliferation and apoptosis, with its effects on apoptosis under different conditions. Aberrant expression of IER3 has been observed in certain cancers, where it regulates tumor cell proliferation, invasion, and metastasis, contributing to tumorigenesis, progression, and prognosis [22]. These functions are linked to its involvement in various signaling pathways, such as mitogen-activated protein kinase/extracellular signal-regulated kinase (MAPK/ERK), PI3K/Akt, and NF-κB pathways [22,23,24]. These signaling pathways play critical roles in osteogenic differentiation. Therefore, we hypothesize that IER3 may regulate osteogenic differentiation in MSCs. Elucidating its role in MSCs’ differentiation could provide a theoretical basis for developing novel therapeutic strategies.

Therefore, this study aims to investigate the expression dynamics of IER3 and osteogenic-related genes during the osteogenic induction of human mesenchymal stem cells (hMSCs) by analyzing publicly available MSCs osteogenic differentiation datasets. To elucidate the functional role of IER3 in hMSCs osteogenesis, an IER3 knockdown cell line will be established. Additionally, RNA–sequencing (RNA–seq) will be utilized to uncover the regulatory mechanisms through which IER3 modulates osteogenic differentiation.

## 2. Materials and Methods

### 2.1. RNA–Seq Analysis of hMSCs

This study utilized two RNA–seq datasets obtained from the GEO database, which consisted of human mesenchymal stem cells (hMSCs) at various time points of osteogenic induction (GSE80614, *n* = 3; GSE185951, *n* = 5). Analysis was performed using the GEO2R online tool, and DEGs were identified through Student’s *t*-test. The criteria for significant differences were set as |log2(FoldChange)| > 2 and *p* < 0.05.

### 2.2. Cell Culture

Commercially acquired, healthy human mesenchymal stem cells (hMSCs) (HUXMD-01001, Oricell, Guangzhou, China) were cultured in alpha-MEM medium (Gibco, Grand Island, NY, USA) supplemented with 10% fetal bovine serum (FBS), 100 U/mL penicillin, and 100 U/mL streptomycin. Adherent cells were trypsinized and passaged after reaching approximately 80% confluence. Cells from passages 3–6 were used for subsequent experiments. Osteogenic and adipogenic induction was initiated when the cells reached 70% confluence. The osteogenic induction medium consisted of alpha-MEM supplemented with 10% FBS, 100 U/mL penicillin, 100 U/mL streptomycin, 10 nM dexamethasone, 10 mM β-glycerophosphate disodium salt, and 50 mg/L Ascorbic acid [25]. The adipogenic induction medium consisted of alpha-MEM supplemented with 10% fetal bovine serum, 100 U/mL penicillin, 100 U/mL streptomycin, 1 μM dexamethasone, 0.2 mM indomethacin, 0.5 mM IBMX, and 0.01 g/L insulin [25].

### 2.3. RNA Extraction, cDNA Synthesis, and Quantitative Real-Time Polymerase Chain Reaction (qRT-PCR)

Total RNA was extracted using TRIzol (Invitrogen, Carlsbad, CA, USA) and assessed for purity using a Nanodrop spectrophotometer (Thermo Scientific, Waltham, MA, USA). Samples that did not meet the purity standards (OD260/OD280 > 1.8 and OD260/OD230 > 1.5) were excluded. Reverse transcription of RNA to cDNA was conducted using a commercial kit (Takara, Kyoto, Japan). Quantitative Real-time PCR was performed with SYBR Green dye (Accurate Biology, Changsha, China) on an Applied Biosystems Q3 system (Thermo Scientific, Waltham, MA USA). Relative gene expression was calculated using the 2^−ΔΔCT^ method. Primer sequences are listed in Table 1.

### 2.4. Lentiviral Transfection of hMSCs for IER3 Knockdown

Lentiviral vectors were constructed to knock down IER3 expression (shIER3) and to serve as negative controls expressing only GFP (shNC). Stable transfected hMSCs were obtained by puromycin selection 72 h post-transfection. Transfection efficiency was assessed via Green fluorescent protein (GFP) fluorescence, and qRT-PCR was used to evaluate gene expression.

### 2.5. ALP and Alizarin Red S (ARS) Staining

After osteogenic induction for 7 to 14 days, IER3 knockdown hMSCs were washed with PBS and fixed with 4% paraformaldehyde (PFA) at room temperature for 15 min. For ALP staining, cells were washed twice with PBS and incubated with ALP staining solution (Beyotime, Shanghai, China) in the dark at room temperature for 5–30 min, followed by washing with ddH_2_O and imaging. ARS staining was performed similarly, using ARS staining solution (Oricell, Guangzhou, China) and incubating for 10 min.

### 2.6. Oil Red O Staining

After adipogenic induction, IER3 knockdown hMSCs were washed with PBS and fixed with 4% PFA for 20 min. Following fixation, cells were immersed in 60% isopropanol, which was then discarded. Freshly prepared Oil Red O staining solution (Solarbio, Beijing, China) was added and stained for 15 min. The excess staining solution was removed, and cells were rinsed with 60% isopropanol until the stroma was clear. The cells were then washed with deionized water and counterstained with Mayer’s Hematoxylin Solution (Solarbio, Beijing, China). After counterstaining, nuclei were cleared with Oil Red O buffer (Solarbio, Beijing, China) before imaging. The area of the red regions, defined by thresholding, was calculated using ImageJ 1.52a (National Institutes of Health, Bethesda, MD, USA).

### 2.7. Ectopic Osteogenesis in Nude Mice

All animal experiments were approved by the Peking University Animal Use and Care Committee (LA2024264). IER3 knockdown cells (shIER3) or negative control cells (shNC) were co-cultured with Bio-Oss collagen (Geistlich, Wolhusen, Switzerland) for 4 h at 37 °C to allow maximal cell attachment. Male BALB/c nude mice (6–8 weeks old) were randomly divided into shIER3 (*n* = 4) and shNC (*n* = 4) groups. After anesthetizing mice with 1% pentobarbital sodium, a 3 cm incision was made on both sides of the dorsal midline, and the Bio-Oss collagen scaffolds with attached cells were implanted subcutaneously. Eight weeks later, mice were euthanized, and implants with surrounding tissues were collected and fixed in 4% PFA for 24 h.

### 2.8. Histological Staining

Implants fixed in paraformaldehyde were subjected to standard paraffin embedding, including dehydration, paraffin infiltration, and sectioning into 5 μm slices. H&E (Solarbio, Beijing, China) and Masson’s trichrome staining (Solarbio, Beijing, China) were performed according to the manufacturer’s protocols. Quantitative analysis was performed on three randomly selected fields per sample at 200× magnification. In H&E staining, newly formed blood vessels were defined as luminal structures densely packed with ≥3 anucleate, biconcave disc-shaped erythrocytes (stained red). The number of newly formed blood vessels was quantified in both shIER3 and shNC groups. For Masson’s trichrome staining quantification, it is necessary to first exclude residual collagen fibers within the Bio-Oss material, which appear as uniformly distributed, sheet-like structures lacking cellular components and exhibit clear boundaries with surrounding cells. Subsequently, the percentage of newly formed collagen area relative to the total tissue area was calculated using ImageJ (National Institutes of Health, Bethesda, MD, USA) [26].

### 2.9. RNA Sequencing and Analysis

Total RNA was isolated from IER3 knockdown hMSCs (shIER3, *n* = 3) and negative control hMSCs (shNC, *n* = 3). Samples were processed for RNA sequencing on the NovaSeq PE150 platform (Magigene, Guangzhou, China). The resulting gene expression matrix underwent rigorous quality control and gene alignment for downstream analysis. DEGs were identified using the DESeq2 package (version 1.42.1) in the R statistical environment, with selection criteria of |log2(FoldChange)| > 2 and *p* < 0.05. DEG analysis included heatmap visualization, GO enrichment analysis, and KEGG enrichment analysis.

### 2.10. Protein Extraction and Western Blot Analysis

Proteins were extracted using RIPA buffer (Solarbio, Beijing, China) supplemented with phosphatase and protease inhibitors (Solarbio, Beijing, China). Protein concentrations were normalized, and samples were stored at −80 °C. Western blot analysis involved loading 20 μg of protein per well alongside a pre-stained molecular weight marker (Thermo Scientific, Waltham, MA, USA) onto 12% Tris gels (Epizyme, Shanghai, China). After electrophoresis, proteins were transferred to PVDF membranes (Merck, Darmstadt, Germany), and immunoblotting was conducted using the e-BLOT system (e-BLOT, Shanghai, China).

### 2.11. Inhibition of the ERK Signaling Pathway

To explore the downstream signaling pathways regulated by IER3, 10 μM U0126 and 20 μM PD98059 (specific MEK1/2 inhibitors, upstream activators of ERK1/2) were added to the osteogenic induction medium. Equal volumes of Dimethyl sulfoxide (DMSO) were used as a control.

### 2.12. Statistical Analysis

Statistical analyses and graph generation were performed using SPSS 26.0 (SPSS, Inc., Chicago, IL, USA) and GraphPad Prism 10.0.0 (GraphPad Software, Boston, MA, USA). Differences between the two groups were analyzed using independent samples *t*-tests and one-way ANOVA. Data are presented as mean ± standard deviation (SD), and a *p* < 0.05 was considered statistically significant.

## 3. Results

### 3.1. Expression of IER3 During Osteogenic Differentiation of hMSCs

**Figure 1:**
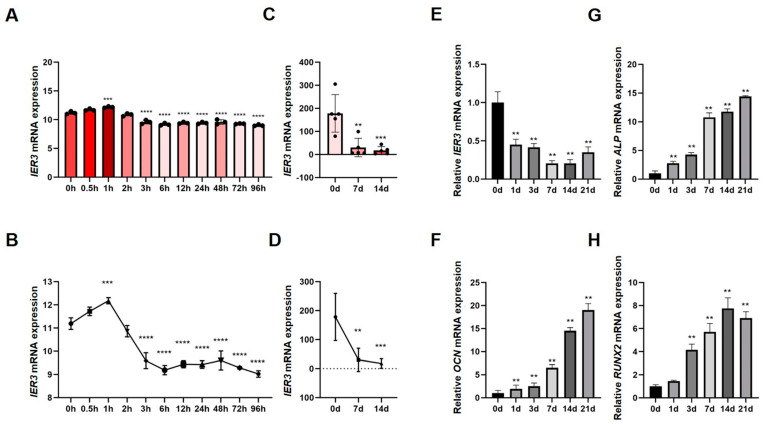
Expression of IER3 and osteogenic makers during osteogenic differentiation of hMSCs. (**A**–**D**) RNA–seq analysis of the GEO datasets showing the expression trend of IER3 during osteogenic induction of hMSCs: (**A**,**B**) IER3 mRNA expression at 96 h of osteogenic induction (RNA–seq data from GSE80614, *n* = 3). (**C**,**D**) IER3 mRNA expression at 14 days of osteogenic induction (RNA–seq data from GSE185951, *n* = 5). (**E**–**H**) Expression levels of IER3 and osteogenic differentiation-related genes during the 21 days of osteogenic induction of hMSCs: (**E**) Relative mRNA expression of IER3 (*n* = 3). (**F**) Relative mRNA expression of OCN (*n* = 3). (**G**) Relative mRNA expression of ALP (*n* = 3). (**H**) Relative mRNA expression of RUNX2 (*n* = 3). Results were normalized to GAPDH expression. **** *p* < 0.0001, *** *p* < 0.001, and ** *p* < 0.01.

Two RNA–seq datasets (GSE80614 and GSE185951) from the GEO database were analyzed. Dataset 1 (GSE80614): RNA–seq analysis of hMSCs during osteogenic induction at multiple time points (0, 0.5, 1, 2, 3, 6, 12, 24, 48, and 96 h) revealed that IER3 expression increased at 1 h after induction and significantly decreased thereafter until day 4 (Figure 1A,B). Dataset 2 (GSE185951): RNA–seq analysis at days 0, 7, and 14 during osteogenic induction showed a continuous and significant decrease in IER3 expression over 14 days (Figure 1C,D). To further investigate IER3 expression during osteogenic differentiation, hMSCs were subjected to osteogenic induction for 21 days. IER3 expression showed significant downregulation during differentiation, reaching its lowest levels on t days 7 and 14, with a slight increase on day 21 (Figure 1E). Osteogenic markers ALP, Osteocalcin (OCN), and RUNX2 were significantly upregulated during differentiation. RUNX2 expression peaked at day 14, while ALP and OCN levels progressively increased throughout the 21-day induction period (Figure 1F–H). The results above show that the expression of IER3 in hMSCs during osteogenic differentiation exhibits a negative correlation with osteogenic-related genes such as ALP, RUNX2, and OCN.

### 3.2. The Effect of IER3 on Osteogenic Differentiation of hMSCs In Vitro

**Figure 2:**
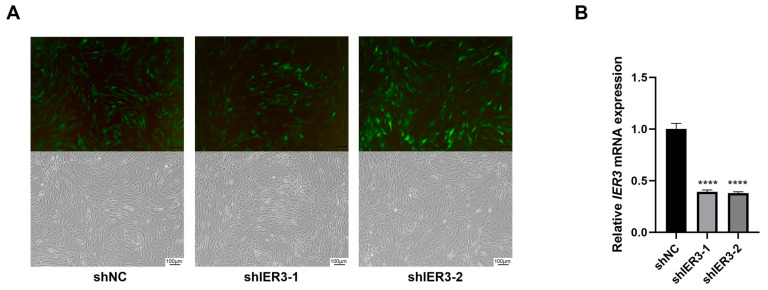
Lentiviral transfection of hMSCs for IER3 knockdown. (**A**) Cell morphology and GFP-positive expression under an inverted microscope and fluorescence microscope at 72 h after hMSCs transfection. (**B**) Efficiency of IER3 knockdown in lentiviral-transfected hMSCs (*n* = 3). Results were normalized to GAPDH expression. **** *p* < 0.0001. Scale bar, 100 μm.

To explore the functional role of IER3 in hMSCs osteogenic differentiation, IER3 was knocked down using lentiviral vectors (shIER3) with shNC as the control. Lentiviral transfection achieved >70% GFP-positive cells after 72 h (Figure 2A), and IER3 mRNA levels were significantly downregulated (~70% knockdown efficiency) (Figure 2B).

**Figure 3:**
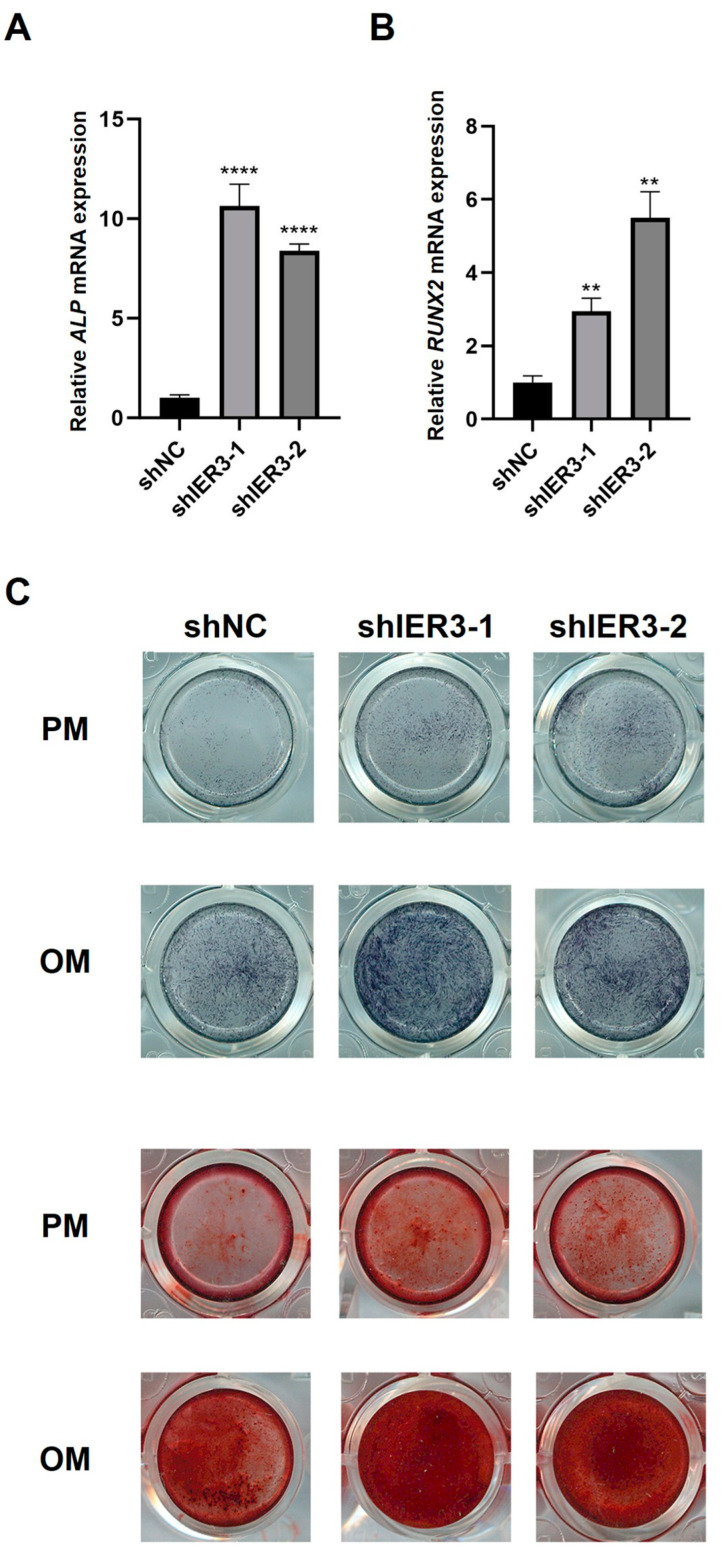
Osteogenic differentiation of IER3 knockdown hMSCs. (**A**) Relative mRNA expression of ALP after IER3 knockdown (*n* = 3). (**B**) Relative mRNA expression of RUNX2 after IER3 knockdown (*n* = 3). Results were normalized to GAPDH expression. **** *p* < 0.0001, ** *p* < 0.01. (**C**) ALP and ARS staining of IER3 knockdown hMSCs under osteogenic induction (*n* = 3). Cells were cultured with either regular proliferation medium (PM) or osteogenic medium (OM).

Under osteogenic induction, ALP and RUNX2 expression levels were significantly higher in shIER3 than in shNC (Figure 3A,B). ALP activity and mineralized nodule formation were markedly enhanced in shIER3 compared to shNC (Figure 3C). The results indicated that the knockdown of IER3 promotes osteogenic differentiation of hMSCs in vitro.

### 3.3. The Effect of IER3 on Osteogenic Differentiation of hMSCs In Vivo

**Figure 4:**
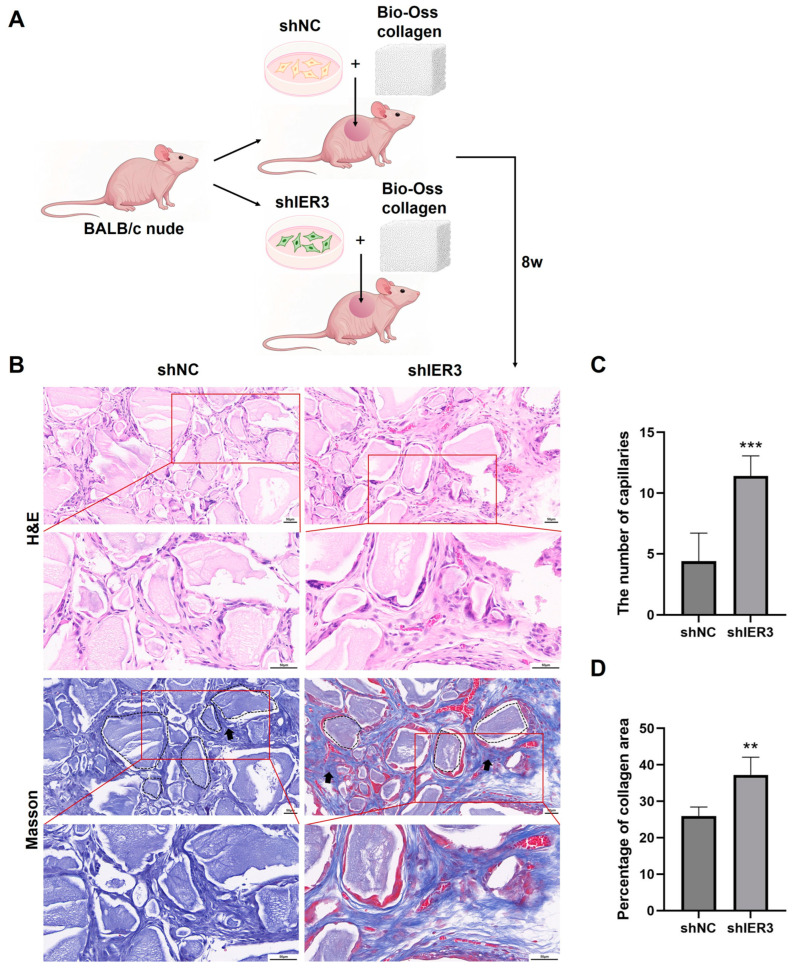
Histological evaluation of IER3 knockdown hMSCs after subcutaneous implantation in nude mice. (**A**) Schematic diagram of hMSCs from the shIER3 and shNC groups, mixed with Bio-Oss collagen and implanted subcutaneously in the backs of nude mice to establish the model (*n* = 4). (**B**) Representative H&E and Masson’s trichrome staining of the implant regions after eight weeks. The black dashed lines indicate representative areas of the residual collagen components of the Bio-Oss collagen, and the black arrows indicate the newly formed collagen. Scale bar, 50 μm. (**C**) The number of capillaries according to the H&E staining images. (**D**) Histomorphometry analysis according to the Masson’s staining images. *** *p* < 0.001, ** *p* < 0.01.

In vivo, shIER3 and shNC cells were mixed with Bio-Oss collagen and implanted subcutaneously in nude mice (Figure 4A). Hematoxylin and eosin (H&E) and Masson’s staining showed that shIER3 implants formed more orderly collagen fibers, with an increased number of blood vessels compared to shNC (Figure 4B–D). The knockdown of IER3 also promotes osteogenic differentiation of hMSCs in vivo.

### 3.4. IER3 Influences Osteogenic Differentiation Through the MAPK/ERK Pathway

**Figure A1:**
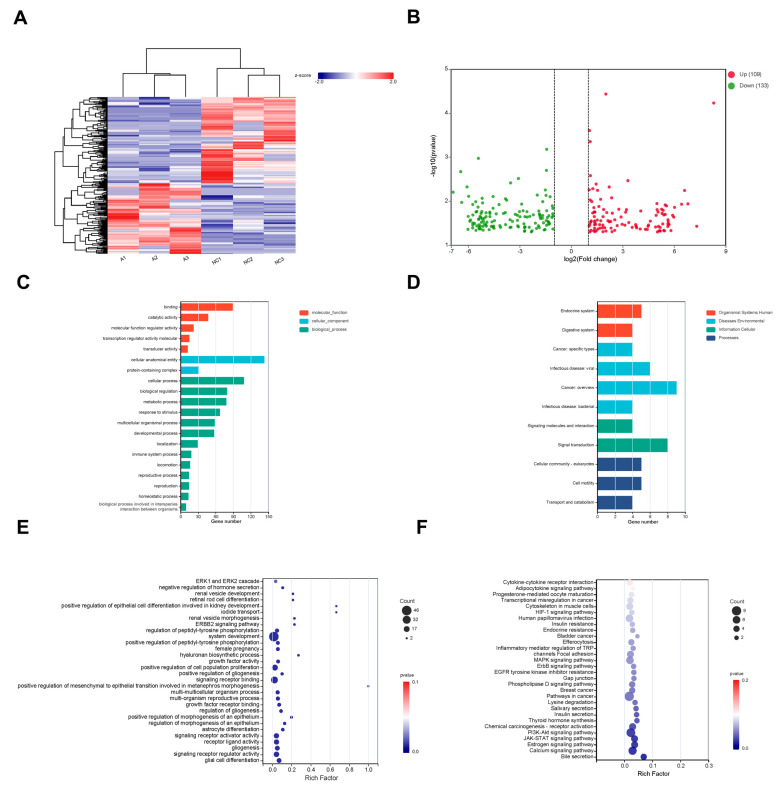
RNA−seq analysis of gene expression differences between shIER3 and shNC. (**A**) Heatmap of differentially expressed genes. (**B**) Volcano plot of differentially expressed genes. (**C**) GO annotation analysis. (**D**) KEGG annotation analysis. (**E**) GO enrichment analysis of differentially expressed genes. (**F**) KEGG enrichment analysis of differentially expressed genes.

RNA–seq analysis of shIER3 and shNC hMSCs revealed 242 differentially expressed genes (DEGs), including 109 upregulated and 133 downregulated genes (Figure A1A,B). GO analysis indicated that DEGs were mainly involved in cellular metabolism, proliferation, and differentiation, with significant participation in the ERK1/2 cascade (Figure A1C,E). KEGG analysis identified pathways such as MAPK and PI3K-Akt among the top 30 enriched pathways (Figure A1D,F).

**Figure 5:**
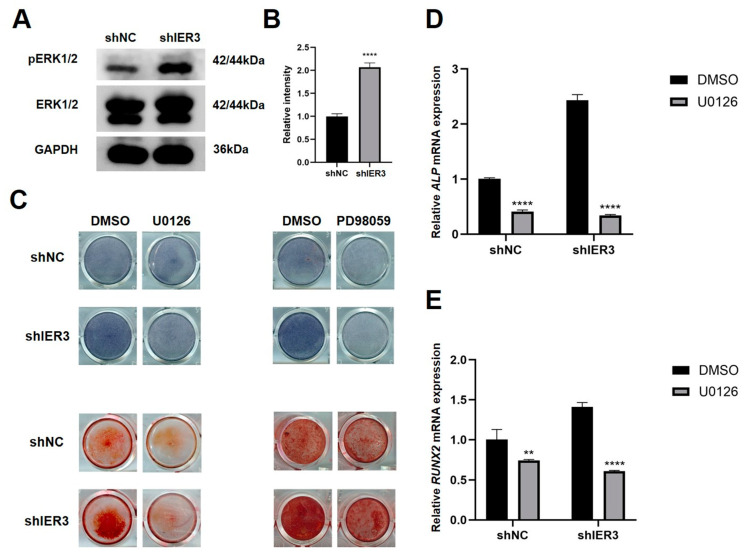
IER3 knockdown regulates osteogenic differentiation of hMSCs through the MAPK/ERK signaling pathway. (**A**,**B**) Expression levels of ERK1/2 and phosphorylated ERK1/2 proteins in IER3 knockdown hMSCs (*n* = 3). Results were normalized to ERK1/2 and GAPDH. (**C**) ALP and ARS staining of IER3 knockdown hMSCs under osteogenic induction with or without MEK1/2 inhibitors (*n* = 3). Cells were cultured with either PM or OM. (**D**) Relative mRNA expression of ALP under U0126 treatment for 7 days (*n* = 3). (**E**) Relative mRNA expression of RUNX2 under U0126 treatment for 3 days (*n* = 3). Results were normalized to GAPDH expression. **** *p* < 0.0001, ** *p* < 0.01.

To validate the involvement of the MAPK/ERK pathway, additional experiments were conducted: Western blot analysis demonstrated significantly increased levels of phosphorylated ERK1/2 protein in shIER3 cells compared to shNC (Figure 5A,B). The role of the ERK pathway in osteogenic differentiation was further assessed using specific inhibitors of MEK1/2, U0126, and PD98059. In the absence of inhibitors, shIER3 cells exhibited significantly higher ALP activity and increased mineralized nodule formation compared to shNC. However, upon treatment with ERK pathway inhibitors, ALP activity and mineralization were reduced in both the shIER3 and shNC groups (Figure 5C). Additionally, with the inhibitor, the mRNA expression of both ALP and RUNX2 was significantly downregulated in both shNC and shIER3 groups (Figure 5D,E). These findings collectively suggest that knockdown of IER3 promotes osteogenic differentiation of hMSCs through modulation of the MAPK/ERK signaling pathway. Inhibition of the MAPK/ERK pathway significantly reduces the osteogenic differentiation potential of IER3 knockdown hMSCs.

### 3.5. The Effect of IER3 on Adipogenic Differentiation of hMSCs In Vitro

**Figure 6:**
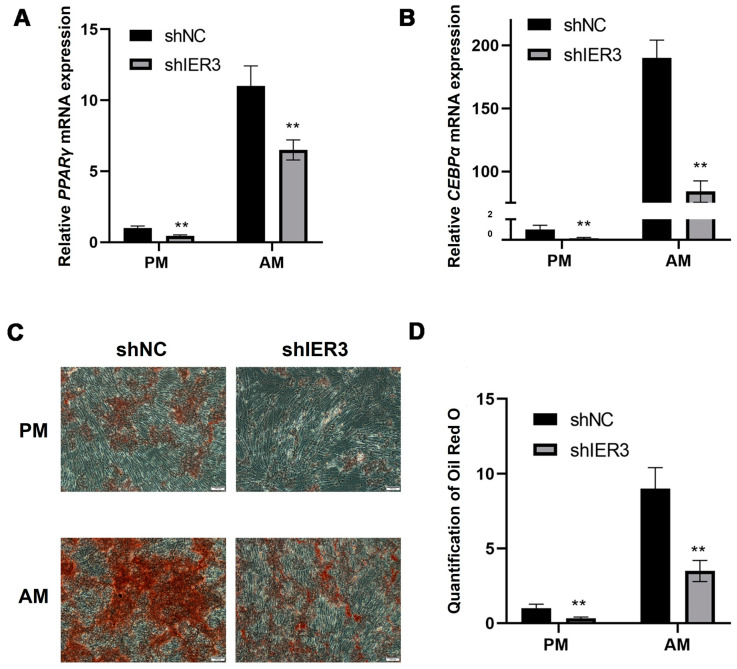
Adipogenic differentiation of IER3 knockdown hMSCs. (**A**) The relative mRNA expression level of PPARγ after IER3 knockdown (*n* = 3). (**B**) The relative mRNA expression level of CEBPα after IER3 knockdown. Results were normalized to GAPDH expression (*n* = 3). (**C**,**D**) Oil Red O staining of IER3 knockdown hMSCs (*n* = 3). Cells were cultured in either proliferation medium (PM) or Adipogenic medium (AM). ** *p* < 0.01. Scale bar, 100 μm.

After culturing in adipogenic medium (AM), the expression levels of peroxisome proliferator-activated receptor gamma (PPARγ) and CCAAT/enhancer-binding protein alpha (CEBPα) in shIER3 were significantly downregulated compared to shNC (Figure 6A,B). Despite this downregulation, both shIER3 and shNC cells cultured in AM formed more lipid droplets compared to those maintained in conventional proliferation medium (PM). Compared to shNC, shIER3 exhibited a significantly higher number of lipid droplets, with the result being statistically significant (Figure 6C,D). These findings indicate that IER3 knockdown inhibits adipogenic differentiation of hMSCs.

## 4. Discussion

IER3, as a gene inducible by various stimuli, has been extensively studied for its role in tumorigenesis and progression [19]. Studies have shown that IER3 is involved in biological processes such as cell proliferation, apoptosis, and tumor cell invasion and metastasis by regulating multiple signaling pathways, some of which are closely related to bone formation [22,23,24]. Therefore, to investigate the regulatory mechanism of IER3 in MSCs osteogenic differentiation at the genetic level, this study first explored gene expression during hMSCs osteogenic differentiation using molecular biology methods. By analyzing RNA–seq data from the GEO database during hMSCs osteogenic differentiation, we found that IER3 was significantly downregulated during osteogenic induction. Further investigation into the relationship between IER3 and osteogenesis-related genes revealed that IER3 exhibited a trend opposite to the gradual upregulation of RUNX2, ALP, and OCN during hMSCs osteogenic differentiation. The downregulation of IER3 may indicate that hMSCs exit the cell cycle and enter the osteogenic differentiation phase.

We successfully constructed an IER3 knockdown hMSCs cell line and further validated its effects. The results showed that ALP activity was significantly increased, and mineralized nodule deposition was markedly enhanced in IER3 knockdown hMSCs, confirming that IER3 knockdown promotes osteogenic differentiation and enhances the mineralization capacity of hMSCs. Concurrently, IER3 knockdown significantly upregulated the expression levels of osteogenesis-related genes RUNX2 and ALP. RUNX2 is a key transcription factor in the early stages of osteogenic differentiation, and elevated ALP is a marker of active osteogenic differentiation [27,28]. These results suggest that IER3 knockdown may promote hMSCs osteogenic differentiation by upregulating the expression of osteogenesis-related genes RUNX2 and ALP, thereby activating osteogenesis-related signaling pathways.

In vivo experiments further validated the promotive effect of IER3 knockdown on hMSCs osteogenic differentiation. Bio-Oss collagen was selected as the biomaterial scaffold for hMSCs due to its proven efficacy in promoting bone formation [29]. In our study, the results revealed that the implants in the IER3 knockdown hMSCs showed increased and more well-organized collagen fibers compared to the negative control group. As the main component of the osteoid matrix, collagen provides the foundation for bone tissue mineralization. The formation of osteoid is a crucial step in bone tissue formation. These results are consistent with our in vitro findings showing upregulated expression of osteogenesis-related genes ALP and RUNX2 after IER3 knockdown, suggesting that IER3 knockdown can improve the osteogenic potential of hMSCs. Additionally, the IER3 knockdown group showed a significant increase in angiogenesis relative to the control group. Previous studies have confirmed that vascular growth and osteogenesis are coupled during skeletal development and bone formation. These results align with prior studies showing that angiogenesis and osteogenesis are coupled processes during skeletal development and bone formation, as effective bone formation depends on concurrent vascularization [30,31]. Newly formed blood vessels can also secrete various growth factors, such as vascular endothelial growth factor (VEGF) and fibroblast growth factor (FGF), to further promote osteogenic differentiation [31]. Therefore, these results indirectly confirm that IER3 knockdown may enhance the in vivo osteogenic differentiation capacity of hMSCs by promoting angiogenesis, thereby playing a positive role in in vivo osteogenesis.

**Figure 7:**
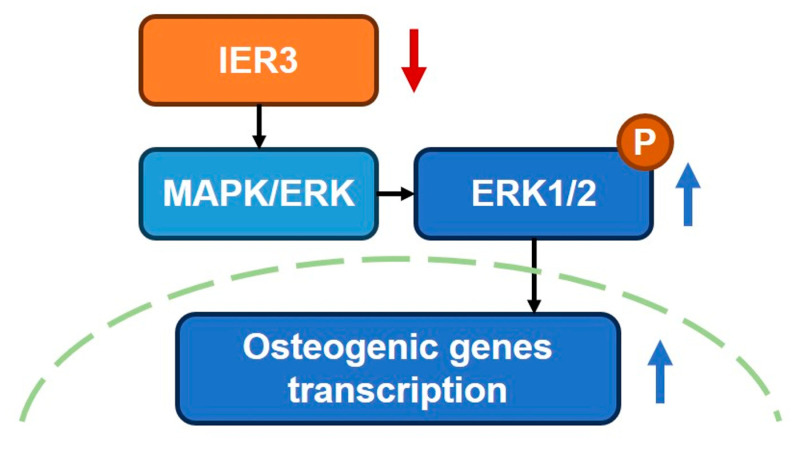
IER3 knockdown regulates the osteogenic differentiation of hMSCs, partially by the MAPK/ERK signaling pathway. IER3 knockdown upregulated the phosphorylation of ERK1/2 and osteogenic genes transcription, thereby promoting the osteogenesis of hMSCs.

To investigate the mechanism by which IER3 regulates osteogenic differentiation in hMSCs, we performed RNA–seq analysis. GO and KEGG enrichment analyses revealed that differentially expressed genes were involved in signaling pathways such as MAPK, PI3K/Akt, and cancer-related pathways, which play important roles in cell proliferation, differentiation, and stress responses. Additionally, GO enrichment analysis indicated that differentially expressed genes were involved in the ERK1/2 cascade, suggesting that IER3 may influence hMSCs osteogenic differentiation through the MAPK/ERK signaling pathway. MAPK includes the ERK1/2, JNK, and p38 MAPK pathways, which are associated with cell proliferation, differentiation, migration, senescence, and apoptosis [32]. ERK1/2 belongs to the MAPK family and functions in signal transduction cascades, transmitting extracellular signals to intracellular targets [33]. Activation of the MAPK/ERK signaling pathway is involved in the early stages of osteogenic differentiation, promoting BMSCs or pre-osteoblasts to enter the osteogenic differentiation state [34,35]. Our results showed that IER3 knockdown significantly upregulated the phosphorylation levels of ERK1/2 proteins in hMSCs, indicating that IER3 knockdown activates the MAPK/ERK signaling pathway. This suggests that IER3 may regulate hMSCs osteogenic differentiation by modulating ERK1/2 phosphorylation and influencing the MAPK/ERK pathway (Figure 7).

To further investigate whether IER3 regulates hMSCs osteogenic differentiation through the MAPK/ERK pathway, we treated IER3 knockdown hMSCs with U0126 and PD98059. U0126 and PD98059 are commonly used inhibitors of the MAPK/ERK pathway that specifically target MEK1/2, thereby preventing ERK1/2 phosphorylation and activation [36,37,38]. We found that U0126 and PD98059 significantly reduced ALP staining and mineralized nodule deposition in the IER3 knockdown group. Our results also showed that ALP and RUNX2 expression was significantly downregulated in the IER3 knockdown group after inhibitor treatment. This indicates that IER3 knockdown may primarily promote hMSCs osteogenic differentiation by affecting ALP and RUNX2 expression through the MAPK/ERK pathway. However, the MAPK/ERK pathway may not be the only mechanism through which IER3 regulates hMSCs osteogenic differentiation. Future studies could further explore the interactions between IER3 knockdown and other signaling pathways.

With aging, MSCs in the bone marrow tend to differentiate into adipocytes, further exacerbating bone metabolism disorders, which is one of the factors influencing the development and progression of osteoporosis [39]. Therefore, regulating the differentiation direction of MSCs and restoring the balance of the bone microenvironment are of great significance for preventing osteoporosis and promoting bone tissue regeneration [40]. Osteogenic and adipogenic differentiation often exhibit mutual inhibition. Between osteogenic and adipogenic lineage commitment and differentiation, a theoretical inverse relationship exists [41]. Thus, we induced adipogenic differentiation in IER3 knockdown hMSCs and found that lipid droplet formation was significantly reduced, indicating that IER3 knockdown inhibits hMSCs adipogenic differentiation. The expression of PPARγ and CEBPα was significantly downregulated after IER3 knockdown. PPARγ and CEBPα are key transcription factors for adipogenic differentiation, regulating the expression of multiple adipogenesis-related genes [42]. IER3 knockdown may block hMSCs adipogenic differentiation by inhibiting the expression of PPARγ and CEBPα. This suggests that IER3 may play a crucial balancing role in determining the fate of hMSCs. Increased ERK1/2 phosphorylation can promote RUNX2 expression and inhibit PPARγ expression in MSCs, thereby promoting osteogenic differentiation and inhibiting adipogenic differentiation [43]. This may also be one of the mechanisms by which IER3 knockdown significantly reduces the adipogenic differentiation capacity of hMSCs. We speculate that under physiological conditions, IER3 may maintain the differentiation balance of hMSCs by regulating the expression of osteogenic and adipogenic-related genes. IER3 knockdown disrupts this balance, causing hMSCs to preferentially differentiate toward the osteogenic lineage. This characteristic of IER3 may provide new therapeutic strategies for bone tissue engineering and regenerative medicine in osteoporosis. However, the mechanisms by which IER3 regulates the multilineage differentiation potential of human mesenchymal stem cells require further in-depth investigation.

## 5. Conclusions

In conclusion, this study highlights the critical role of IER3 in regulating the osteogenic differentiation of hMSCs. Our findings demonstrate that IER3 knockdown enhances osteogenic differentiation, as evidenced by increased expression of key osteogenic markers, elevated ALP activity, and mineralized nodule formation, both in vitro and in vivo. Also, IER3 knockdown was found to promote angiogenesis, a process closely coupled with bone formation. IER3 knockdown regulates the osteogenic differentiation of hMSCs by the MAPK/ERK signaling pathway, and it also inhibits adipogenic differentiation. These results position IER3 as a potential molecular target for the development of innovative therapies to address osteoporosis and bone defect-related diseases.

## Figures and Tables

**Table 1:** Primer Sequences for qRT-PCR.

Gene	Primer (5′–3′)	
ALP	Forward	AGCCCTTCACTGCCATCCTGTAT
	Reverse	CGCCTGGTAGTTGTTGTGAGCAT
RUNX2	Forward	CCGCCTCAGTGATTTAGGGC
	Reverse	GGGTCTGTAATCTGACTCTGTCC
OCN	Forward	CACTCCTCGCCCTATTGGC
	Reverse	CCCTCCTGCTTGGACACAAG
GAPDH	Forward	GGTCACCAGGGCTGCTTTTA
	Reverse	GGATCTCGCTCCTGGAAGATG
IER3	Forward	CAGTCGAGGAACCGAACCC
	Reverse	GATCTGGCAGAAGACGATGGT
CEBPα	Forward	TAGGATAACCTTGTGCCTTGGAAAT
	Reverse	GTCTGCTGTAGCCTCGGGAA
PPARγ	Forward	GCCTGCATTTCTGCATTCTG
	Reverse	CACGGAGCTGATCCCAAAG

## Data Availability

The original contributions presented in the study are included in the article, further inquiries can be directed to the corresponding authors.

## Abbreviations

The following abbreviations are used in this manuscript:

ALP	Alkaline phosphatase
AM	Adipogenic medium
ARS	Alizarin Red S
CEBPα	CCAAT/enhancer-binding protein alpha
DEGs	Differentially expressed genes
DMSO	Dimethyl sulfoxide
ERK	Extracellular regulated protein kinases
H&E	Hematoxylin and eosin
hMSCs	Human mesenchymal stem cells
IER3	Immediate Early Response 3
MAPK	Mitogen-activated protein kinase
MSCs	Mesenchymal stem cells
OCN	Osteocalcin
OM	Osteogenic medium
PFA	Paraformaldehyde
PM	Proliferation medium
PPARγ	Peroxisome proliferator-activated receptor gamma
RNA–seq	RNA sequencing
RUNX2	Runx-related transcription factor 2
